# Histologic Analysis of Transjugular Liver Biopsy Specimens for Early Prediction of Prognosis in Acute Liver Failure

**DOI:** 10.1016/j.gastha.2022.02.017

**Published:** 2022-04-05

**Authors:** Toru Ishikawa, Kazuki Ohashi, Erina Kodama, Takamasa Kobayashi, Motoi Azumi, Yujiro Nozawa, Akito Iwanaga, Tomoe Sano, Terasu Honma

**Affiliations:** 1 Department of Gastroenterology, Saiseikai Niigata Hospital, Niigata, Japan; 2 Department of Nursing, Sapporo University of Health Sciences, Hokkaido, Japan; 3 Faculty of Health Sciences, Hokkaido University, Hokkaido, Japan

**Keywords:** Acute Hepatic Failure, Outcome, Histopathology, Massive Hepatic Necrosis, Predictor

## Abstract

**Background and Aims:**

The prognosis of acute liver failure (ALF) treated with conservative therapy is extremely poor. Histologic diagnosis at the time ALF occurs provides important clues for determining the prognosis, including indications for liver transplant. Transjugular liver biopsy (TJLB), which helps clarify the pathology of ALF, may be an effective clinical parameter that contributes to prognosis prediction, including indications for liver transplant.

**Methods:**

In this prospective study, 79 patients who underwent TJLB with ALF were enrolled between May 2002 and March 2021. The relationships between prognosis and the extent of tissue necrosis on TJLB specimens, serum parameters related to the cause, and clinical parameters were investigated.

**Results:**

Model for end-stage liver disease-sodium, hepatic encephalopathy predicting, total bilirubin, hepatocyte growth factor, ammonia, coma rate, and histologic diagnosis were identified as prognostic factors on univariate analysis. Histologically, 13 of 16 patients with massive hepatic necrosis died or had a liver transplant. On multivariate analysis, the only prognostic factor was massive hepatic necrosis. There were no treatment-related complications, and TJLB was technically successful in all patients.

**Conclusion:**

In diagnosing the cause of ALF and understanding its pathology, TJLB contributes to predicting the prognosis of ALF based on histologic findings together with liver function tests and imaging findings, and it is an important diagnostic technique for determining diagnostic and treatment eligibility, including indications for liver transplant. When determining the best timing for patient selection and liver transplant, the finding of massive hepatic necrosis on TJLB specimens at the time ALF occurred was the most important prognostic factor.

## Introduction

Acute liver failure (ALF) is a clinical syndrome associated with various causes that bring rapid loss of hepatocellular function. It is usually related to coagulation disorders and encephalopathy and has a high mortality rate.^1^^,^^2^ The treatment of ALF includes intensive care incorporating artificial liver support with plasmapheresis and continuous hemodiafiltration. Even so, the mortality rate is high, and the timing of a transition to liver transplant (LT) is also important.^3^^,^^4^ LT is an innovative treatment option for ALF, and it has decreased deaths in several thousand high-risk patients.^5^, ^6^, ^7^

Therefore, early prediction of the outcomes of ALF patients at risk of death if LT is not performed and of patients who are expected to survive with intensive care is necessary.

The establishment of an early-stage diagnostic method that helps evaluate the need for LT in ALF, in addition to a serological approach, is urgently needed.^8^^,^^9^

Liver biopsy is normally done percutaneously for diagnosing chronic hepatitis in general but also in cases of hepatic dysfunction of unknown cause. However, in cases of liver disease of unknown cause, there is a risk of unexpected bleeding and other complications. Moreover, in patients who present with bleeding diathesis, such as those with ascites and those with advanced coagulation disorders, percutaneous liver biopsy is generally contraindicated.^10^^,^^11^ With transjugular liver biopsy (TJLB), it is possible to safely sample liver tissue even in patients with advanced liver damage.^12^, ^13^, ^14^, ^15^

Histologic examination of liver biopsy specimens obtained by TJLB can be used together with the severity of liver damage to identify and evaluate the cause.

Prognostic models such as the model for end-stage liver disease (MELD), hepatic encephalopathy (HE) predicting model, and the Japanese scoring system are also used to predict the mortality rate of patients.^16^, ^17^, ^18^, ^19^

However, few studies have evaluated the usefulness of TJLB in terms of whether diagnosing the state of liver tissue necrosis at the time ALF occurs is an early predictor of outcome in ALF patients.

The main objective of this prospective study was to investigate the usefulness of TJLB in terms of whether hepatic tissue from TJLB at the time ALF occurs can be a prognostic factor for ALF patients.

## Materials and Methods

This prospective observational study enrolled 79 patients with ALF who underwent TJLB between May 2002 and March 2021. The inclusion criteria for enrollment were as follows: (1) initial onset without a diagnosis of chronic hepatitis or cirrhosis; (2) alanine aminotransferase (ALT) >200 IU/L and/or aspartate aminotransferase (AST) >200 IU/L; and (3) prothrombin time (PT) and international normalized ratio (PT-INR) >1.5 or PT activity <40%.^20^ In TJLB, the right internal jugular vein is punctured under local anesthesia, an 8-Fr sheath is inserted, and the morphology of the vein is confirmed with a 6-Fr balloon catheter, after which the biopsy is performed. A cutting needle from a liver access and biopsy set (Cook) and an aspiration biopsy needle jointly developed with Hakko Co, Ltd, were used. Biochemical tests, computed tomography imaging, and TJLB were performed on the day of occurrence along with diagnosis in all patients. In this study, a comprehensive evaluation, including the results of biochemical tests, was conducted to identify independent prognostic factors.

All patients received intensive care, including artificial liver support consisting of plasmapheresis immediately after TJLB. LT was performed in accordance with the Japanese scoring system to predict the prognosis of ALF.^18^ The analysis compared 65 survivors and 14 nonsurvivors, defined as patients who died or underwent LT because of lack of response to intensive care including artificial liver support.

The histologic diagnosis of TJLB was stratified as spotty/focal necrosis, zonal necrosis, submassive necrosis, and massive necrosis following the classifications of Krishna.^21^

Written informed consent was obtained from all patients in advance. The study was conducted in conformance with the principles of the Declaration of Helsinki. All protocols followed in this study were approved by the Institutional Review Board of Saiseikai Niigata Hospital (approval number: E05-13).

### Statistical Analysis

Data are expressed as medians (25th–75th percentiles) or n (%). Participants were classified as survivors and nonsurvivors (those who died or underwent LT within 30 days), and then comparisons between groups were made with the Mann-Whitney U test and Fisher’s exact test, as appropriate. Variables that were significant on univariate analysis were entered into a multiple logistic regression analysis to identify the factors associated with prognosis, including death and LT. All statistical analyses were performed with EZR version 1.42.^22^ A *P* value of <.05 was considered significant.

## Results

The patients’ baseline characteristics are shown in Table 1. Of the 79 patients, 48 were men, and 31 were women. The median age at the time of diagnosis was 55.0 years.

**Table 1:** Clinical Features and Laboratory Data of the Study Cohort on Admission

Variable	Overall (n = 79)
Sex, male (%)	60.7
Age (y)	55.0 (41.0–68.5)
Etiology (viral/nonviral)	20/59
Viral (HAV/HBV)	20 (3/17)
AIH	21
DILI	17
Alcohol	7
Unknown	14
MELD	15.0 (10.5, 21.0)
MELD-Na	17.00 (14.00, 22.00)
HE predicting	34.19 (23.99, 55.24)
T-Bil (mg/dL)	4.18 (1.94, 11.52)
AST (U/L)	1053.00 (316.50, 2558.50)
ALT (U/L)	971.00 (266.00, 2176.00)
Cre (mg/dL)	0.74 (0.59, 1.02)
PT-INR	1.52 (1.42, 1.89)
HGF (ng/mL)	1.12 (0.56, 2.32)
NH_3_ (μg/dL)	59.00 (37.50, 76.50)
Coma (%)	31.6
Pathology	
Focal/spotty	12 (15.2%)
Zonal	40 (50.6%)
Submassive	11 (13.9%)
Massive	16 (20.3%)

DILI, drug-induced liver injury.

The etiologies of underlying ALF were hepatitis A infection, hepatitis B infection, autoimmune hepatitis, drug-induced liver injury, alcohol intake, and unknown origin in 3 (3.8%), 17(21.5%), 21(26.6%), 17(21.5%), 7(8.9%), and 14 patients (17.7%), respectively.

All of hepatitis B infection group were diagnosed hepatitis B surface antigen and anti-HBc IgM become positive, supporting an acute hepatitis B virus (HBV) diagnosis, these patients were treated by nucleotide analog. Initiation of corticosteroid therapy may be considered for patients with autoimmune hepatitis.

Artificial liver support with plasmapheresis and/or hemodiafiltration was performed for most of the comatose patients.

The MELD score was 15.0, MELD-Na 17.0, HE predicting 34.19, total bilirubin (T-Bil) 4.18 mg/dL, AST 1053 U/L, ALT 971 U/L, PT-INR 1.52, and hepatocyte growth factor (HGF) 1.12 ng/mL. Histologically, focal/spotty necrosis was seen in 12 patients (15.2%), zonal necrosis in 40 patients (50.6%), submassive necrosis in 11 patients (13.9%), and massive necrosis in 16 patients (20.3%).

A comparison of the clinical features on admission between nonsurvivors and survivors is presented in Table 2. There were 14 nonsurvivors, of whom 12 died, and 2 underwent LT.

**Table 2:** Clinical Features and Laboratory Data of the Study Cohort on Admission by Outcome

Variable	Died/LT (n = 14)	Survivors (n = 65)	*P* value
Sex, male (%)	42.9	64.6	.15
Age (y)	63.0 (54.0, 68.0)	55.0 (40.0, 69.0)	.19
Etiology (viral/nonviral)	2/12 (14.3/85.7)	18/47 (27.7/72.3)	.50
Etiology (viral/AIH/ DILI/alcohol/unknown)	2/4/3/1/4	18/17/4/6/10	.75
MELD	19.5 (10.0, 25.75)	14.0 (11.0, 20.0)	.30
MELD-Na	22.50 (17.50, 27.50)	17.00 (13.00, 22.00)	.02
HE predicting	39.84 (32.11, 74.31)	34.09 (23.45, 50.49)	.07
T-Bil (mg/dL)	11.50 (1.69, 17.52)	4.08 (2.00, 9.45)	.37
AST (U/L)	245.00 (140.25, 1405.00)	1154.00 (466.00, 2782.00)	.06
ALT (U/L)	251.50 (52.50, 714.75)	1162.00 (486.00, 2512.00)	<.01
Cre (mg/dL)	0.68 (0.58, 0.99)	0.74 (0.61, 1.03)	.71
PT-INR	1.81 (1.44, 2.27)	1.48 (1.42, 1.83)	.08
HGF (ng/mL)	3.46 (1.46, 4.20)	0.84 (0.55, 1.51)	<.01
NH_3_ (μg/dL)	80.50 (59.25, 146.25)	55.00 (37.00, 68.00)	.015
Coma (%)	64.3	24.6	<.01
Pathology			<.01
Focal/spotty	0	12	
Zonal	0	40	
Submassive	1	10	
Massive	13	3	

AIH, autoimmune hepatitis; DILI, drug-induced liver injury.

Of the etiology, there were 20 viral patients, 3 from hepatitis A virus (HAV) and 17 from HBV.

Of the 14 nonsurvivors who died or underwent LT, 64.3% lapsed into a coma during the course, vs 24.6% of survivors who lapsed into a coma; this difference was significant.

The viral origin was HBV in 2 nonsurvivors, one of whom underwent LT and one who died.

No significant differences were seen between the survivors and nonsurvivors in age, sex, etiology, MELD score, total/direct Bil ratio, AST, creatinine, or PT-INR, but significant differences were seen in MELD-Na, ALT, HGF, NH_3_, and pathology.

The pathologic results for the survivors and nonsurvivors are shown in Figure. All patients in the focal/spotty necrosis and zonal necrosis groups survived.

**Figure:**
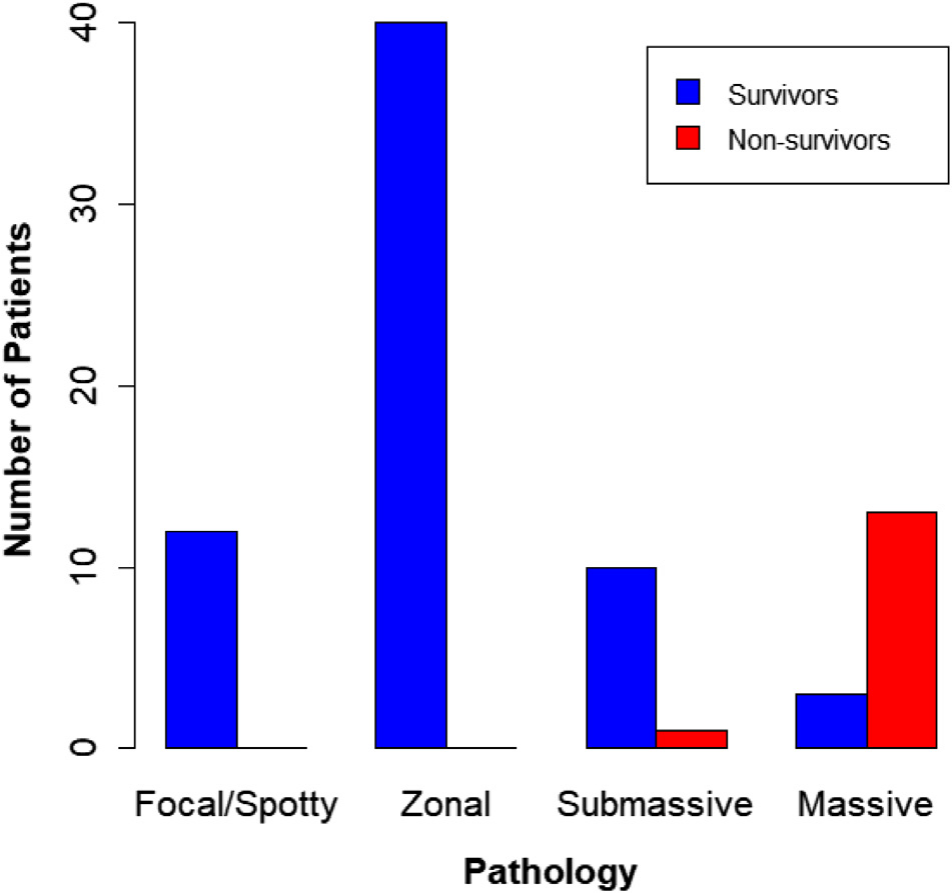
Histologic findings of transjugular liver biopsy specimens of acute liver failure patients according to survival status.

On univariate analysis, MELD-Na (*P* = .04), HE predicting (*P* = .03), T-Bil (*P* = .02), HGF (*P* = .03), NH_3_ (*P* < .01), coma rate (*P* < .01), and pathology (massive necrosis; *P* < .01) were identified as factors related to nonsurvival.

On multivariate analysis using a logistic regression model, only pathology (massive necrosis) was identified, with an odds ratio of 1250.0 (95% confidence interval: 21.2–73400.0; *P* < .01; Table 3).

**Table 3:** Predictive Factors Associated With Nonsurvivors

Variable	Univariate analysis		Multivariate analysis	
	OR (95% CI)	*P* value	OR (95% CI)	*P* value
Sex (male)	0.41 (0.13–1.33)	.14		
Age	1.03 (0.99–1.07)	.16		
Etiology (viral/nonviral)	0.44 (0.09–2.14)	.31		
MELD	1.04 (0.98–1.10)	.21		
MELD-Na	1.07 (1.00–1.13)	.04	0.94 (0.75–1.17)	.56
HE predicting	1.03 (1.00–1.06)	.03	0.98 (0.89–1.07)	.63
T-Bil	1.10 (1.01–1.19)	.02	1.27 (0.95–1.69)	.10
AST	1.00 (1.00–1.00)	.46		
ALT	1.00 (1.00–1.00)	.26		
Cre	1.10 (0.77–1.56)	.60		
PT-INR	1.30 (0.93–1.82)	.13		
HGF	1.17 (1.02–1.35)	.03	1.15 (0.73–1.81)	.54
NH3	1.02 (1.01–1.04)	<.01	0.99 (0.96–1.03)	.70
Coma (%)	5.51 (1.61–18.9)	<.01	2.16 (0.14–33.3)	.58
Pathology (massive)	269.0 (25.9–2790.0)	<.01	1250.0 (21.2–73400.0)	<.01

CI, confidence interval; OR, odds ratio.

The tissue lengths of the specimens obtained by TJLB were 16.2 ± 7.1 (14–26) mm, the number of portal triads was 6.8 ± 1.5, and there was no tissue fragmentation. All specimens obtained were adequate for making a diagnosis (100%).

The TJLB success rate was 100%, and no serious complications were seen.

## Discussion

In this prospective study, 79 patients with ALF who underwent diagnostic TJLB were evaluated, and its utility as a prognostic indicator was examined. The main causes of ALF are viruses and drugs, but autoimmune disorders have also come to be included recently. The pathology of ALF also includes many other causes besides those mentioned earlier. A prognostic formula for ALF with higher sensitivity and specificity and a higher correct diagnosis rate for criteria indicating LT are needed. The development of treatments other than LT, including antiviral therapy, that will lead to higher survival rates is also urgently needed. Eligibility for LT in these patients needs to be determined rapidly. When considering diagnosis and treatment of this condition, a treatment strategy based on histologic findings is also thought to be necessary. The purpose of this study was to evaluate whether TJLB to determine the pathology of ALF can be used for predicting prognosis, including the indications for LT.

TJLB can be done even in cases when percutaneous liver biopsy is contraindicated because of coagulation disorders, ascites, ALF, excessive adipose tissue, LT, or other conditions^23^^,^^24^ and is recognized as an alternative biopsy method in these contraindicated cases.

Dotter reported TJLB in dogs in 1964,^25^ Hanafee reported transjugular percutaneous cholangiography in 1967,^26^ and Weiner and Hanafee^27^ reported the first clinical application of TJLB in 1970. Rösch et al^23^ later began full clinical use of TJLB in 1973 and published many reports. In TJLB, a biopsy needle punctures the liver parenchyma from the hepatic vein side via a catheter placed in the hepatic vein and collects tissue. TJLB is a diagnostic technique that enables the making of important diagnoses in patients with ascites and those in whom percutaneous liver biopsy is contraindicated because of bleeding diathesis. However, the usefulness of TJLB for ALF has not been fully investigated.

The subjects in the present study were ALF patients who underwent TJLB. The etiology was viral in 20 of these patients (3 HAV and 17 HBV) and nonviral in 59. The viral cause in 2 nonsurvivors was HBV: one patient underwent LT and one died. However, in 15 survivors with HBV, ALF due to HBV was quickly diagnosed from serological and histologic findings, and a nucleotide analog was administered.

Of the 14 nonsurvivors who died or underwent LT, 64.3% lapsed into a coma during the course, which was significantly higher than the percentage of survivors who lapsed into a coma (24.6%). Quick control of encephalopathy is important for improving the prognosis, but the patients with poor encephalopathy control progressed significantly more to necrosis on TJLB specimen evaluation.

Hepatic coma may be clinically recognized by systemic hypertension and bradycardia, decerebrate posturing or rigidity, conjugated eye movements, and loss of pupillary reflex. Serial computed tomography of the head is also monitoring the diagnosis of cerebral edema.

Various forms of continuous renal replacement therapy are preferred for patients with ALF, as these therapies avoid the large metabolic and hemodynamic fluctuations associated with intermittent dialysis, which can precipitate episodes of raised intracranial pressure.^2^^,^^28^

Combination therapy involving continuous hemodiafiltration, plasma exchange and the infusion of fresh frozen plasma are typically performed to support liver functions, such as detoxification and supplementation of coagulation factors. ALF patients are treated using a combination of these treatments.^29^

Significant differences were seen between survivors and nonsurvivors in MELD-Na, AST/ALT ratio, ALT, HGF, NH_3_, and pathology. In the groups with focal/spotty necrosis or zonal necrosis on TJLB, all patients survived, and it is thought that such patients can be saved with multimodal intensive care treatment.

Factors for nonsurvival were identified as MELD-Na, HE predicting, T-Bil, HGF, NH_3_, coma (%), and pathology (massive necrosis). On multivariate analysis, only pathology (massive necrosis) was identified as a factor, with an odds ratio of 1250.0 (95% confidence interval: 21.2–73400.0). In patients with a TJLB diagnosis of massive necrosis, transplant should be the first choice to save the patient.

At the same time, many cases of tissue fragmentation with TJLB have been reported, and fragmentation may make histologic diagnosis difficult.^30^ The ALF patients in the present study had evident coagulation disorders and poor general conditions, but the TJLB success rate was 100%, and no serious complications were seen. TJLB had a high level of safety even in ALF patients, and a definitive diagnosis was made from the collected tissue. TJLB can contribute to the determination of an appropriate treatment plan in all cases and the prediction of nonsurvival.

In determining the best timing for patient selection for LT, TJLB findings at the time ALF occurs may be the most effective prognostic factor.

This study has several limitations. First, the sample size was small. Confirmation of these findings will require a larger prospective clinical trial. Second, it is not clear whether TJLB reflects the histologic state of the entire liver.

Third, excessive consumption of acetaminophen is a main cause of ALF in developed countries. In the present study, however, there were no patients in whom excessive consumption of acetaminophen was the cause of ALF. Finally, the data were from a single center, and selection bias cannot be ruled out.

In conclusion, the results of the present study suggest that histologic findings of TJLB specimens at the time ALF occurs reflect the severity of liver damage and the prognosis in ALF patients. TJLB can be used as an accurate prognostic biomarker in early ALF, and it is beneficial in clinical decision-making.

TJLB is a useful technique for diagnosing the cause of ALF and understanding its pathology. Together with liver function tests and imaging findings, TJLB contributes to predicting the prognosis of ALF patients from the histologic findings, and it may assist in improving diagnostic performance related to the indications for LT.
